# Development and Content Validity of the Physical Activity Questionnaire-Young Children (PAQ-YC) to Assess Physical Activity in Children between 5 and 7 Years

**DOI:** 10.3390/healthcare9060655

**Published:** 2021-05-31

**Authors:** Marta Amor-Barbosa, Montserrat Girabent-Farrés, Ferran Rosés-Noguer, Anna Ortega-Martínez, Almudena Medina-Rincón, Caritat Bagur-Calafat

**Affiliations:** 1 Physiotherapy Department, Universitat Internacional de Catalunya, 08195 Barcelona, Spain; mamor@uic.es (M.A.-B.); aortegam@uic.es (A.O.-M.); cbagur@uic.es (C.B.-C.); 2 Physiotherapy Department, School of Health Sciences, TecnoCampus-Pompeu Fabra University, Mataró, 08302 Barcelona, Spain; almumrincon@hotmail.com; 3 Paediatric Cardiology Department, Vall d’Hebron University Hospital, 08035 Barcelona, Spain; froses@vhebron.net; 4 Paediatric Cardiology Department, Royal Brompton and Harefield NHS Foundation Trust, London SW3 6NR, UK; 5 Physiotherapy Department, Fundació Aspace Catalunya, 08001 Barcelona, Spain; 6 Rehabilitation Section, Centro Hospitalario Pere Virgili, 08023 Barcelona, Spain

**Keywords:** physical activity, questionnaire, content validity, paediatrics

## Abstract

Childhood is a critical period in the development and consolidation of healthy habits, such as the practice of physical activity (PA). It is essential to have valid instruments to measure PA from an early age. The aim of this study was to design and evaluate the content validity of the Physical Activity Questionnaire-Young Children (PAQ-YC) to measure the PA level in children aged 5–7 years. The first version of the questionnaire was tested by a 2-round Delphi study. It was established as a consensus criterion that the relative interquartile range (RIR) and/or the coefficient of variation (CV) were ≤20%. The most significant discrepancies in the Delphi survey (n = 11–13) were observed for items about hours of Physical Education or similar activities at school (item 7: RIR = 20, CV = 38.73) and for items about participation in Physical Education (item 8: RIR = 25, CV = 15.45). The cognitive interviews (n = 5) confirmed the version agreed by the experts. The results show that the PAQ-YC presents adequate content validity in terms of relevance, comprehensiveness and comprehensibility.

## 1. Introduction

Physical activity (PA) is a fundamental basis in chronic disease prevention, and it is related to numerous benefits apart from physical health, such as psychological well-being or cognitive performance. Solid evidence demonstrates that PA is associated with a reduced risk of increasing weight and adiposity [1,2,3] and favourable signs in bone health, such as bone structural strength, bone mineral content and area [4,5,6] in preschoolers. For children and adolescents, it has been proved that regular PA is related to better cardiorespiratory resistance [7,8,9,10,11], better muscular resistance and strength [12,13,14,15], decreased weight and corporal fat percentage [16,17,18], better bone health [17,19,20,21], less risk of cardiovascular and metabolic diseases [11,22,23], reduction of depressive symptoms or anxiety [24,25,26,27] and an improvement on cognitive indicators, such as memory, processing velocity, attention and academic performance [28,29,30]. Considering the impact of sedentary conduct on the health of children and adolescents, evidence suggests that the higher percentage of sedentarism, the less cardiovascular and metabolic health and higher adiposity index [31,32,33].

Childhood is a critical period in developing and consolidating healthy habits since a child’s brain is sensitive to being influenced by the environment in its modifiable conduct [34]. Therefore, it is essential to have valid instruments capable of objectifying the level of PA performed from an early age. Even though some authors consider that doubly labelled water [35], calorimetry [36] or direct observation [37] are the gold standards in measuring levels of PA in children, others defend that there is no real gold standard [38,39,40,41,42,43]. Scientific evidence suggests that any current method can capture all the components of PA, and the selection decision can only rely on objective criteria depending on practicality, psychometric properties and the aim of the investigation [41,44,45,46].

Compared to direct methods, questionnaires offer some advantages, such as low cost and associated time of distribution and administration, and the possibility of gathering information from many participants [45,47]. Another strength is that they can identify dimensions and domains of PA [38,44] and have no risk of affecting usual patterns of PA because the reference period to measure the PA is previous to their administration [40,48]. However, a large number of available questionnaires for the paediatric population and the lack of high-quality studies make it difficult to establish recommendations about the best questionnaire available [49,50,51,52,53,54,55,56].

In response to the global need for comparable measures of PA within and between countries, an initiative started in 1996 to develop a compilation of valid questionnaires that culminated in the integration of an International Consensus Group that designed a total of eight versions of the International Physical Activity Questionnaire (IPAQ). The aim of this group was to consolidate a line of indirect measures that could monitor and guide the development of policies focused on PA related to health. Since then, this set of questionnaires has been expanded with the creation of versions focused on other populations and with adaptations for different countries and cultures [57,58]. Therefore, the interest continues with the study of the instruments of the IPAQ group, specifically focusing on the questionnaires available for preschoolers and children. There are various adapted versions of IPAQ questionnaires for different ranges of age in paediatrics. On the one hand, the version for children from 8 to 14 years old has been published and named Physical Activity Questionnaire-Children (PAQ-C) [59,60]. Besides, the English version of the questionnaire for children under 5 years old, known as the Early Years Physical Activity Questionnaire (EY-PAQ), is to be answered by parents [61]. Nevertheless, there is no adapted version for children aged 5–7 years old. Therefore, this study aimed to design the Physical Activity Questionnaire-Young Children (PAQ-YC) to evaluate the PA level in children aged 5–7 years old and evaluate its content validity in terms of face validity, relevance, comprehensiveness and comprehensibility.

## 2. Materials and Methods

The study of the development of the Physical Activity Questionnaire-Young Children (PAQ-YC) was conducted following the directives of design and analysis of the Consensus-based Standards for the Selection of Health Measurement Instruments guideline (COSMIN) [62].

### 2.1. Description of the Construct

PAQ-YC aims to measure the total level of PA done by a child aged 5–7 years old in a typical week through the school term. It includes PA at school (Physical Education or other similar activities and break times), transport and leisure time (after-school activities, active games at home and indoor equipment, and outdoor sports) during the last 7 days, assuming that they can be understood as a representation of a typical week unless an extraordinary event could have occurred and could have prevented the fulfilment of the daily activities. Additionally, two questions related to sedentary behaviour during leisure time have been included.

The PAQ-YC design was adjusted to its supposed application with discriminative purposes, with the intention that the questionnaire results helped to identify active enough and inactive subjects, following the international recommendations on PA [63]. On its development, the time of administration no longer than 15 min was considered, and the answers given by parents, children and teachers were unified in a unique version.

### 2.2. Selection of the Items to Obtain the Final Version

The PAQ-YC design was based on the conceptual framework defined by Pettee Gabriel KK et al. [41], with its adaptations for the paediatric population. The method used to choose the items was preceded by a complete revision of the literature that allowed a compilation of questions susceptible to be included in the final version. The development of the PAQ-YC took as a reference the published versions for different ranges of age and the indications of the literature regarding the type of PA usually done by children from 5 to 7 years old. The PAQ-C version validated for the Spanish population by Manchola-González et al. for children ranged 8–14 years old [64], and the EY-PAQ version validated by Bingham et al. for children less than five years old [61] were used as a model. The final title for the PAQ-YC was chosen to emphasise the targeted range of age.

Later, the answer options for each item were described. For questions about leisure time and transport, a grid format including predefined time ranges was used. For the items referred to school, a 3-item scale, in which the respondent had to mark the closest to the usual conduct, was defined. Regarding its structure, there were two parts: questions where parents could answer by themselves (part 1) and questions that needed the participation of the child or teacher to confirm the answers given (part 2).

A Delphi survey was created to evaluate the content validity. The proposal of collaboration was sent to potential experts related to PA in paediatrics. To evaluate the suitability of the selected experts, the knowledge coefficient (Kc) was used, obtaining their expertise level by calculating the weighted average of the punctuation obtained in each of the items shown in Table 1. Kc was calculated regarding the information that the expert showed about the topic of research. Experts that did not reach the demandable critical level of 0.8 were excluded [65].

Afterward, the questionnaire consultation rounds were done with the members of the panel of experts included. An online survey was elaborated to generate a debate that allowed ordered feedback. The consultation rounds suggested an iterative process and were repeated as often as necessary until an agreement was reached. The identity of the participants was anonymous.

The experts were asked for expressing their level of agreement with each of the items, as well as the general characteristics of the questionnaire to evaluate its face validity. The level of agreement was expressed in a rating scale from 1 to 5, in which 1 was the lowest level possible “strongly disagree”, 2 for “disagree”, 3 for “indifference”, 4 for “agree”, and 5 for “strongly agree”. Moreover, a separate section for comments was enabled to allow the participants to manifest specific improvement suggestions.

For each consultation round, the median (Me) and quartiles 1 (Q1) and 3 (Q3) of the answers obtained were calculated. It was established as a consensus criterion that the relative interquartile range (RIR = (Q3 – Q1/Me) x 100) and/or the coefficient of variation (CV = (SD/Me) × 100) were less or equal to 20% [66].

Once the consensual version by the experts was obtained, a process of cognitive interviews in a representative sample of parents was performed. This study received the approval of the Ethical Committee of the Universitat Internacional de Catalunya (Code: FIS-2018-06). First, they were given the paper questionnaire, and they were asked to self-report it. Then, a semi-structured interview was done, where they were asked about the relevance, comprehensiveness and comprehensibility of each of the items and the general characteristics. Lastly, the spontaneously mentioned issues were commented. A verbal report was the approach used. Notes were written during the process.

## 3. Results

The first version of the questionnaire was evaluated by the Delphi survey. The final panel was comprised of 13 experts (7 men, 6 women, mean age 38.85 years (11.43); Kc = 8.67 (0.7)) on the first round and 11 experts (5 men, 6 women, mean age 39.91 years (11.99); Kc = 8.50 (0.61)) on the second round. In the first round, 2 Physiotherapists, 2 graduated in Sports Science, 2 Physiotherapists and Graduated in Sports Science, 4 Primary School teachers, 1 Preschooler teacher and 1 Preschooler teacher and Psychologist participated. In the second round, two of the Primary School teachers that participated in the first round did not participate in the second one.

Two rounds were needed to reach the general agreement. In the first round, the experts answered “strongly agree” or “agree” on items 1–5 of the first part, on the representativity of a usual week item and the general characteristics of the questionnaire. In the second round, experts answered “strongly agree” or “agree” on items 1–5 of part 1, on items 9 and 10 of part 2, on the item about representativity of a usual week and on the general characteristics of the questionnaire. It is essential to mention that on items 6 and 8, only one expert did not answer “strongly agree” or “agree”, whilst 2 experts showed disagreement on item 7 compared to the general opinion (Table 2).

The second part questions and question 7 of part 1 were motives of discrepancies, showing a RIR and CV higher than 20%. However, a general agreement could be considered due to the accomplishment of at least one of the established criteria (item 6: RIR = 20.00 and CV = 15.45; item 7: RIR = 20.00 and CV = 38.73; item 8: RIR = 25.00 and CV = 15.45). Table 3 shows the degree of the agreement for each of the items and general characteristics in the consulting rounds.

Once the consented version by experts was done, the questionnaire was submitted to a process of cognitive interviews in a total of 5 heterogeneous families regarding sex (3 boys, 2 girls, 5 mothers, 2 fathers), age (children 5.8 (0.84) years old; parents 36 (5) years old), parent’s level of education [67] (1 Lower Secondary School, 1 Upper Secondary School, 1 Professional Training, 4 University education or equivalent) and demographic environment (2 in a village between 5.000–10.000 inhabitants, 1 in a town between 10.000–20.000 inhabitants and 4 in a city of more than 100.000 inhabitants). It is important to mention that both parents were interviewed separately in two families, so there were 7 adults interviewed.

The responsible investigator (MA) was familiar with the use and validation of questionnaires in paediatrics [68,69]. Moreover, she was trained in developing PA questionnaires addressed to the general infantile population. The interviews took between 30 and 40 min. Improvement suggestions were not very relevant and were mainly related to explanations in the definition of the questions. No specific approach was used to evaluate the collected information because the suggestions made by the families were simple and did not specify the need for making essential changes. Moreover, as the modifications were not significant, it was considered that there was not a lack of essential aspects on the construct, and the developing process of the PAQ-YC was finished. The final version of the PAQ-YC is accessible in the supplementary material.

## 4. Discussion

The first version of the PAQ-YC was designed through a selection and reduction of items process and was tested by an online 2-round Delphi survey among experts in physical activity and a representative sample of the target population. The results showed adequate content validity in terms of face validity, relevance, comprehensibility and comprehensiveness of the items and the clarity of the general characteristics.

Regarding the construct, PAQ-YC pretends to capture the total PA done in all intensity ranges and in all domains. At this juncture, among the available questionnaires for the range of age of interest in this study, the great variety of constructs evaluated stand out. For example, the Netherlands Physical Activity Questionnaire (NPAQ) was designed to evaluate the preferences of activities assuming an association with the levels of PA [70,71], meanwhile the Harro’s questionnaire [72,73], the Assessment of Young Children’s Activity using Video Technology (ACTIVITY) questionnaire [74] and the South American Youth/Child Cardiovascular and Environment Study Physical Activity (SAYCARE) questionnaire [75] quantify the total PA so that they could be similar to PAQ-YC. On the other side, questionnaires that include at least two years of the age range of the population of PAQ-YC (5–6 years old or 6–7 years old) gather more delimited constructs, focusing on the evaluation of a higher intensity PA. For example, the Children’s Leisure Activities Study Survey (CLASS) questionnaire [76], the School Health Action, Planning and Evaluation System (SHAPES) questionnaire [77], the Bringolf-Isler questionnaire [78], the Canadian Health Measures Survey (CHMS) questionnaire [79] or the England Health Survey [80] inform about moderate to vigorous PA. Compared to the questionnaires included in the IPAQ group for paediatric ages, EY-PAQ [61], PAQ-C [59,60] and Physical Activity Questionnaire-Adolescents (PAQ-A) [81] evaluate the PA in the context of moderate to vigorous intensity, unlike PAQ-YC, that measures PA in all the different levels of intensity.

With reference to the recall period, PAQ-YC goes back to the last 7 days. In PA questionnaires aimed for the same or close range of age, recall periods are very different. For example, NPAQ inquires about the PA of the previous 6 months [70,71]; EY-PAQ gathers activities of the last month [61]; the SAYCARE questionnaire [75], the England Survey of Health of Physical Activity [80]; SHAPES questionnaire [77], PAQ-C [59,60] and PAQ-A [81] refer to the last 7 days; Harro’s questionnaire [72,73] and ACTIVITY questionnaire [74] evaluate the PA of the previous day, and Bringolf-Isler questionnaire gathers two typical days [78]. Experts point out that the answers depend on recall bias to define activity patterns [40,44,82], especially complex in paediatrics due to the flashing characteristic of PA [37,83]. About the “typical” notion, there is disagreement among experts regarding its adequacy, although some authors consider that the word creates confusion [57,84].

Another critical point in the initial formulation of the items of PAQ-YC was the decision of quantifying PA in terms of type, duration, frequency and intensity. Building this part was extremely difficult due to the sporadic behaviour of children’s PA, which makes this activity challenging to remember, quantify and classify [37].

For items 1–6, an approach based on categories of activity with an answers table that pretends to gather detailed information about frequency and duration was used. Concerning the type of activity, offering closed lists was ruled out because the variety of PA done by children makes it difficult to have a moderate-extension inventory without dismissing any significant action [85]. Nevertheless, examples of activities included in every item were given to facilitate remembrance and clarify the type of activities asked in each question [86]. About the format of other questionnaires for the same or similar range of age, some use the same approach as a checklist, like NPAQ [70,71], CLASS [76], Bringolf-Isler questionnaire [78], or EY-PAQ [61], while others classify PA in categories, as Harro’s questionnaire [72,73], ACTIVITY [74], SAYCARE [75], CHMS [79] or SHAPES [77]. Finally, PAQ-C [64] and PAQ-A [87] combine both formats, as they show items for activity categories and a 22-activity checklist. Regarding questionnaires that classify PA in categories and PAQ-YC, it is usual that they clarify which type of activities they are referring to in each item [72,73,79]. In PAQ-C [59,60] and PAQ-A [81], only some of the questions give concrete examples of activities.

The duration was expressed in reduced intervals in part 1, while the frequency was gathered for every day of the week. For part 2, options of answers were based on a closed scale. Activities during the school period are developed as usual behaviour and have little variability, so the opportunity for PA remains constant and can be estimated [88,89,90]. Questionnaires for the same or similar range of age differ from PAQ-YC on the format and detailed expression to gather duration and frequency. For example, the original version of NPAQ does not consider duration nor frequency [70], whilst it includes one item about time spent watching television on its modified version [71]; Harro’s questionnaire [72,73], SAYCARE [75] and CLASS [76] gather the duration and frequency for every activity; ACTIVITY divides the day into 6 periods, and registers the activities done in each period [74]; SHAPES indicate the number of hours and the increasing of 15 min for every day of the week on its questions [77], while CHMS [79] and Bringolf-Isler questionnaire [78] only ask for information about the duration of each activity, with similar intervals as PAQ-YC. EY-PAQ [61] is also like the first part of PAQ-YC. Nevertheless, the manner of quantifying frequency differs sustainably from it, as it asks for the number of days of the week that each activity is done, while PAQ-YC is supposed to be filled every day. It is important to mention that PAQ-C [59,60] and PAQ-A [81] do not ask for information of duration in any case. In this context, the graph format of item 9 of PAQ-C or item 8 of PAQ-A is like the design of the options of answers in the first part of PAQ-YC.

Regarding the intensity, the average of the energetic cost for every activity category was calculated following the Youth Energy Expenditure Compendium [91]. For the questionnaires commented on previously, the way that intensity is evaluated differs depending on the format. For example, NPAQ does not gather the intensity of the activities on the checklist [70,71]; Harro’s questionnaire splits activities from low to moderate intensity and activities from moderate to vigorous intensity [72,73]; SHAPES [77] and SAYCARE [75] define intensity in relative terms; ACTIVITY uses videoclips from 3 to 5 s that show levels of intensity [74]; CLASS [76] classifies the activities of the checklist in moderate or high intensity allocating MET values from the Adult Compendium [92], unlike PAQ-YC, that uses the references from the Youth Energy Expenditure Compendium [93]; while in Bringolf-Isler [78] and CHMS [79] PA is included in the moderate-to-vigorous category. Gathering intensity in relative terms was excluded because the effort perception depends on individual characteristics [94]. It was also considered to be inappropriate to express intensity only in terms of effort, such as sweating, increasing cardiac frequency or dyspnoea [95,96]. Therefore, and even though it is admitted that the measurement error always occurs, scientific literature shows that it can be minimised by describing intensity in absolute terms [47,84].

Once the first version of the PAQ-YC was designed, the content validity study began through a double-consultation process [97,98]. The process of consulting experts and representative samples are relevant when creating any questionnaire but are more important when the instrument is based on a formative model, as PA questionnaires [99].

The process of selection of the experts represents the focal point of the survey. In our study, we decided to establish different items to calculate a median average according to the importance (Kc). We did not consider any external criterion because, being a heterogeneous group, inconsistency could occur. Other authors, such as Cabero-Almenara suggests leaning on the expert self-report, as well as on the sources that allow increasing the answer given [65,100]. On the other hand, the COSMIN group, in its international Delphi study, establish as an expertise criterion to have at least 5 publications on PubMed about the area of interest [98]. Regarding the number of experts, it was considered a number between 10 and 15, provided that the necessary heterogeneity could be ensured to approach the aim of the consulting through the composition of the group. For example, Ruiz-Olabuénaga and Ispuzua suggest a number between 10 and 30 [101]; García and Fernández between 15 and 25 [102], while others suggest that for operativity issues, it is not recommended to have more than 50 experts [103]. Next, the agreed version of the PAQ-YC with the panel of experts was submitted to a cognitive interviews process in a representative sample of the population. Due to the lack of agreement, the principle of saturation was prioritised [86,104] over the number of interviewees, even though it was decided to recruit at least 7 participants. Some authors suggest that between 7 and 10 interviews are enough [105,106,107], while others defend that a large sample is needed [108]. It is recommended to establish it depending on the variability of the population characteristics and the key aspects of the construct [86].

Regarding the quantitative information collection method, the semi-structured interview was used and followed the same structure as on the Delphi survey to ensure a comprehensive and ordered approach but allowing certain flexibility to adapt the interview to the families’ concerns. The election of the semi-structured interview was justified by the need of exploring the issue deeply over focal groups, which main strength is their capacity to identify a variety of inter-individual experiences and perspectives. In addition, it has been documented that some individuals may be reluctant to express their point of view in front of a group so that some information could be lost [86].

The approach used to interrogate participants was a verbal report. While it is true that more approaches are valid, some authors consider that a verbal report is the most appropriate way to evaluate the participant’s familiarity with the issue and the terminology used. Furthermore, it has been recognised that the interviewer may obtain certain information that otherwise the participant would not give [86,107,109].

Although some authors recommend transcribing records of the interviews to collect data, on the cognitive validation study of PAQ-YC, the decision was only to take notes. This decision was made based on three reasons. First, one family expressed inconvenience in being recorded during the interview. For that, the COSMIN guideline recommends taking notes when participants do not feel comfortable when recording [97]. Secondly, literal transcription methods are designed to document more complex interviews, usually performed in focus groups [110]. Nevertheless, interviews performed for PAQ-YC were made individually, so the process was more straightforward, and the notes taken were enough. Thirdly, the recording could bring problems related to data protection policy [86].

Furthermore, to analyse data collected during interviews, some authors recommend using complex methods designed for procedures with a larger number of participants and a larger flow of information [110]. Instead of this, in agreement with Brod et al. [86], data analysis was done iteratively, beginning as soon as first opinions about the questionnaire were obtained.

Once the interviewing process was finished, the content validity study of the PAQ-YC was terminated [99]. As the contributions made by the population were not very relevant, the commented issues were included without re-evaluating the final version [84]. With regard to the lack of major contributions to the questionnaire, we think it was due to the exhaustive phase of obtaining concepts. COSMIN recognises that the risk in avoiding important aspects is low when the previous phase to the item reduction and experts’ consulting is done carefully [84].

## 5. Limitations

The main limitation of this study is related to the difficulty of designing questionnaires for the 5 to 7-year age group. It includes a population between Pre-Primary Education and Primary Education, that combines times when parents are not in charge of their children, but children do not have enough resources to answer the questionnaire by themselves [37,111,112]. A parent-reported questionnaire was opted for in PAQ-YC, pretending to abridge the provided information by children or teachers at school. This decision was taken based on the difficulty of parents in reporting the PA done when they are not there [113,114] and on that the expert’s recommendations about self-reporting say that it should be only applied beyond 10 years old [49,114,115,116,117].

## 6. Conclusions

The findings of development and content validity of the PAQ-YC show that it is adequate in terms of relevance, comprehensiveness and comprehensibility of the items.

## Figures and Tables

**Table 1:** Knowledge coefficient (Kc) ponderation.

**Evaluation Items**	**Ponderation**
Knowledge of PA in educational centres in Spain	0.20
Knowledge of PA done by children at school period	0.20
Practical experience in PA in school stage	0.25
Knowledge about recommendations of PA in school stage	0.25
Abilities in using instruments to evaluate the level of PA	0.10

**Table 2:** Response rates for each of the items and general characteristics.

**Variable**	**Round 1**					**Round 2**				
**% of Agreement**	**1**	**2**	**3**	**4**	**5**	**1**	**2**	**3**	**4**	**5**
Item 1				38	62					100
Item 2				31	69				36	64
Item 3				23	77					100
Item 4				23	77				9	91
Item 5				15	85				9	91
Item 6		8	8	15	69			9	27	64
Item 7		8	15	31	46	18			27	55
Item 8		8	31	38	23			10	45	45
Item 9			31	31	38				18	82
Item 10			24	38	38				36	64
Representativity of a usual week				23	77				9	91
Clarity of the introductory content				31	69				18	82
Comprehension easiness				38	62				9	91
Clarity and simplicity of the format				54	46				36	64
Division in two parts				23	77				9	91
Number of questions				23	77				9	91
Content appropriateness				31	69				18	82

1 “strongly disagree”, 2 “disagree”, 3 “indifference”, 4 “agree”, 5 “strongly agree”, dashed line: separation between part 1 and 2.

**Table 3:** Degree of the agreement for each of the items and general characteristics in the consulting rounds.

	**Variable**	**Min**	**Max**	**Q1**	**Me**	**Q 3**	**RIR**	**SD**	**Mean**	**CV**
Round 1	Item 1	4	5	4	5	5	20	0.51	4.62	10.97
	Item 2	4	5	4.75	5	5	5	0.48	4.69	10.24
	Item 3	4	5	5	5	5	0	0.44	4.77	9.19
	Item 4	4	5	5	5	5	0	0.44	4.77	9.19
	Item 5	4	5	5	5	5	0	0.38	4.85	7.75
	Item 6	2	5	4	5	5	20	0.97	4.46	21.68
	Item 7	2	5	3.75	4.5	5	27.78	0.99	4.15	23.76
	Item 8	2	5	3	4	4	25	0.93	3.77	24.59
	Item 9	3	5	3	4	5	50	0.86	4.08	21.15
	Item 10	3	5	3.75	4	5	31.25	0.8	4.15	19.27
	Representativity of a usual week	4	5	5	5	5	0	0.44	4.77	9.19
	Clarity of the introductory content	4	5	4.75	5	5	5	0.48	4.69	10.24
	Comprehension easiness	4	5	4	5	5	20	0.51	4.62	10.97
	Clarity and simplicity of the format	4	5	4	4.5	5	22.22	0.52	4.46	11.63
	Division in two parts	4	5	4.75	5	5	5	0.44	4.77	9.19
	Number of questions	4	5	5	5	5	0	0.44	4.77	9.19
	Content appropriateness	4	5	4.75	5	5	5	0.5	4.7	10.24
Round 2	Item 1	5	5	5	5	5	0	0	5	0
	Item 2	4	5	4	5	5	20	0.5	4.64	10.88
	Item 3	5	5	5	5	5	0	0	5	0
	Item 4	4	5	5	5	5	0	0.3	4.91	6.14
	Item 5	4	5	5	5	5	0	0.3	4.91	6.14
	Item 6	3	5	4	5	5	20	0.69	4.55	15.13
	Item 7	1	5	4	5	5	20	1.55	4	38.73
	Item 8	3	5	4	4	5	25	0.67	4.36	15.45
	Item 9	4	5	5	5	5	0	0.4	4.82	8.4
	Item 10	4	5	4	5	5	20	0.5	4.64	10.88
	Representativity of a usual week	4	5	5	5	5	0	0.3	4.91	6.14
	Clarity of the introductory content	4	5	5	5	5	0	0.4	4.82	8.4
	Comprehension easiness	4	5	5	5	5	0	0.3	4.91	6.14
	Clarity and simplicity of the format	4	5	4	5	5	20	0.5	4.64	10.88
	Division in two parts	4	5	5	5	5	0	0.3	4.91	6.14
	Number of questions	4	5	5	5	5	0	0.3	4.91	6.14
	Content appropriateness	4	5	5	5	5	0	0.4	4.82	8.4

Min: minimum, Max: maximum, Q1: quartile 1, Me: mean, Q3: quartile 3, RIR: Relative Interquartile Range, SD: standard deviation, CV: Coefficient of Variation, dashed line: separation between part 1 and 2

## Data Availability

The data presented in this study are available on request from the corresponding author. The data are not publicly available due to privacy reasons.

## Supplementary Materials

The following are available online at https://www.mdpi.com/article/10.3390/healthcare9060655/s1.
