# Supplementary material for: Development and Content Validity of the Physical Activity Questionnaire-Young Children (PAQ-YC) to Assess Physical Activity in Children between 5 and 7 Years
Source: Healthcare (Basel). 2021 May 31;9(6):655. doi: 10.3390/healthcare9060655 (PMC8230329; doi:10.3390/healthcare9060655)
Supplement: Supplementary file 1 [file healthcare-09-00655-s001.zip › healthcare-1223911-supplementary.pdf]

Supplementary material:

Cuestionario de Actividad Física para niños/as entre 5 y 7 años

Fecha: ……………………..………

Nombre y apellidos del niño/a: …………………………………………………………………………………………………..……………

Edad del niño/a: ……….… años ……….. meses

Sexo del niño/a: Masculino ☐ Femenino ☐

Curso escolar: ……………………………..

Parentesco con el niño/a: Padre ☐ Madre ☐ Tutor/a ☐

Queremos saber cuánta actividad física ha hecho su hijo/a en los últimos 7 días, con qué frecuencia y durante cuánto tiempo. Esto incluye todas las actividades como deportes, gimnasia, danza… que le hacen sudar o sentirse cansado, o juegos que hacen que se acelere su respiración como saltar a la comba, correr, trepar y otros.

El cuestionario está formado por dos bloques:

- El bloque 1 contiene preguntas que hacen referencia a las actividades que su hijo/a realiza en su tiempo libre. Estas preguntas podrá responderlas usted solo/a.
- El bloque 2 contiene preguntas relacionadas con la actividad física que su hijo/a hace en la escuela. Para responder a las preguntas del bloque 2, le pedimos que se siente con su hijo/a y que le pregunte para marcar la respuesta. Si no estuviera seguro de la respuesta, le rogamos que consulte la información con los profesores de la escuela.

Le agradecemos total sinceridad en sus respuestas. La información que nos proporcione será tratada confidencialmente y de forma anónima.

Recuerde:

– Marque con una **X** la casilla que se corresponda con su respuesta
– Debe responder a todas las preguntas. No hay preguntas correctas o incorrectas

PREGUNTAS DEL BLOQUE 1

Actividad física en el tiempo libre

1. En los últimos 7 días, ¿qué días y durante cuánto tiempo diría usted que su hijo/a ha hecho actividades dirigidas por monitor/entrenador en horario extraescolar que requieran estar activo (por ejemplo, practicando deportes, danza…)? No tenga en cuenta en esta pregunta el tiempo en la escuela, como el recreo o la Educación Física.

Marque con una **X** la casilla que se corresponda con su respuesta o escriba el número de horas y minutos en caso de que sean más de 2 horas.

Si su hijo/a ha hecho más de una actividad extraescolar el mismo día, sume el tiempo que ha dedicado a cada actividad para obtener el tiempo total. *Por ejemplo minutos.: el lunes ha ido 1 hora a natación y 45 minutos a patinaje. El tiempo total es: 1 hora + 45 minutos = 1 hora y 45*

	Nada o menos de 10 minutos	Entre 11 y 30 minutos	Entre 31 y 59 minutos	Entre 1 hora y 1 hora y 29 minutos	Entre 1 hora y media y 2 horas	Más de 2 horas (escribir cuánto)
Lunes						…… horas y …… minutos
Martes						…… horas y …… minutos
Miércoles						…… horas y …… minutos
Jueves						…… horas y …… minutos
Viernes						…… horas y …… minutos
Sábado						…… horas y …… minutos
Domingo						…… horas y …… minutos

2. En los últimos 7 días, ¿qué días y durante cuánto tiempo diría usted que su hijo/a ha jugado activamente en casa (por ejemplo, bailando, corriendo, empujando juguetes, jugando a videojuegos activos como la Nintendo Wii…) o en instalaciones de juego interiores (por ejemplo, en una ludoteca, en una piscina de bolas…)?

Marque con una **X** la casilla que se corresponda con su respuesta o escriba el número de horas y minutos en caso de que sean más de 2 horas.

Si su hijo/a ha hecho más de una actividad el mismo día, sume el tiempo que ha dedicado a cada actividad para obtener el tiempo total. *Por ejemplo: el lunes ha jugado en la ludoteca 30 minutos y en casa ha bailando mientras escuchaba música durante 20 minutos. El tiempo total es 30 minutos + 20 minutos = 50 minutos.*

	Nada o menos de 10 minutos	Entre 11 y 30 minutos	Entre 31 y 59 minutos	Entre 1 hora y 1 hora y 29 minutos	Entre 1 hora y media y 2 horas	Más de 2 horas (escribir cuánto)
Lunes						…… horas y …… minutos
Martes						…… horas y …… minutos
Miércoles						…… horas y …… minutos
Jueves						…… horas y …… minutos
Viernes						…… horas y …… minutos
Sábado						…… horas y …… minutos
Domingo						…… horas y …… minutos

3. En los últimos 7 días, ¿qué días y durante cuánto tiempo al día diría usted que su hijo/a ha jugado activamente en exteriores (por ejemplo, en el jardín, en el parque…)? No tenga en cuenta las actividades extraescolares dirigidas que se han incluido en la pregunta 1 ni el tiempo de recreo en la escuela.

Marque con una **X** la casilla que se corresponda con su respuesta o escriba el número de horas y minutos en caso de que sean más de 2 horas.

Si su hijo/a ha hecho más de una actividad el mismo día, sume el tiempo que ha dedicado a cada actividad para obtener el tiempo total. *Por ejemplo: el lunes ha jugado en el parque 30 minutos y en casa ha jugado en el jardín durante 20 minutos. El tiempo total es 30 minutos + 20 minutos = 50 minutos.*

	Nada o menos de 10 minutos	Entre 11 y 30 minutos	Entre 31 y 59 minutos	Entre 1 hora y 1 hora y 29 minutos	Entre 1 hora y media y 2 horas	Más de 2 horas (escribir cuánto)
Lunes						...... horas y ...... minutos
Martes						...... horas y ...... minutos
Miércoles						...... horas y ...... minutos
Jueves						...... horas y ...... minutos
Viernes						...... horas y ...... minutos
Sábado						...... horas y ...... minutos
Domingo						...... horas y ...... minutos

Transporte activo

4. En los últimos 7 días, ¿qué días y durante cuánto tiempo diría usted que su hijo/a se ha desplazado de un sitio a otro de forma activa (por ejemplo, caminando, en bicicleta, en patinete…) sin utilizar ningún medio de transporte (ni coche, ni autobús, ni metro…)? Incluya en esta pregunta si el niño/a va caminando a la escuela y otros posibles desplazamientos (por ejemplo, ir de compras, ir a las actividades extraescolares…).

Marque con una **X** la casilla que se corresponda con su respuesta o escriba el número de horas y minutos en caso de que sean más de 2 horas.

Si su hijo/a se ha desplazado varias veces al día sin utilizar ningún medio de transporte, sume el tiempo que ha tardado en desplazarse cada una de las veces para obtener el tiempo total. *Por ejemplo: el lunes ha ido y vuelto del colegio caminando (10 minutos de trayecto de ida y 10 minutos de trayecto de vuelta) y después ha ido y vuelto al parque en bicicleta (15 minutos de trayecto de ida y 15 minutos de trayecto de vuelta). El tiempo total es 10 minutos + 10 minutos + 15 minutos + 15 minutos = 50 minutos.*

	Nada o menos de 10 minutos	Entre 11 y 30 minutos	Entre 31 y 59 minutos	Entre 1 hora y 1 hora y 29 minutos	Entre 1 hora y media y 2 horas	Más de 2 horas (escribir cuánto)
Lunes						...... horas y ...... minutos
Martes						...... horas y ...... minutos
Miércoles						...... horas y ...... minutos
Jueves						...... horas y ...... minutos
Viernes						...... horas y ...... minutos
Sábado						...... horas y ...... minutos
Domingo						...... horas y ...... minutos

Comportamiento sedentario en el tiempo libre

5. En los últimos 7 días, ¿qué días y durante cuánto tiempo diría usted que su hijo/a ha estado sentado jugando con juguetes o sin moverse haciendo actividades como colorear, dibujar, hacer manualidades, hacer los deberes…? No tenga en cuenta las horas en la escuela ni el tiempo con dispositivos electrónicos (por ejemplo, la TV, el ordenador, los videojuegos...).

Marque con una **X** la casilla que se corresponda con su respuesta o escriba el número de horas y minutos en caso de que sean más de 2 horas.

Si su hijo/a ha hecho más de una actividad el mismo día, sume el tiempo que ha dedicado a cada actividad para obtener el tiempo total. *Por ejemplo: el lunes ha coloreado durante 15 minutos y ha hecho los deberes durante 1 hora. El tiempo total es 15 minutos + 1 hora = 1 hora y 15 minutos.*

	Nada o menos de 10 minutos	Entre 11 y 30 minutos	Entre 31 y 59 minutos	Entre 1 hora y 1 hora y 29 minutos	Entre 1 hora y media y 2 horas	Más de 2 horas (escribir cuánto)
Lunes						...... horas y ...... minutos
Martes						...... horas y ...... minutos
Miércoles						...... horas y ...... minutos
Jueves						...... horas y ...... minutos
Viernes						...... horas y ...... minutos
Sábado						...... horas y ...... minutos
Domingo						...... horas y ...... minutos

6. En los últimos 7 días, ¿qué días y durante cuánto tiempo diría usted que su hijo/a ha estado sentado o sin moverse haciendo actividades como ver la TV, hacer los deberes con ordenador o Tablet, jugar con un videojuego, jugar al ordenador, móvil o Tablet? No tenga en cuenta las horas en la escuela ni los videojuegos activos como la Nintendo Wii.

Marque con una **X** la casilla que se corresponda con su respuesta o escriba el número de horas y minutos en caso de que sean más de 2 horas.

Si su hijo/a ha hecho más de una actividad el mismo día, sume el tiempo que ha dedicado a cada actividad para obtener el tiempo total. *Por ejemplo: el lunes ha visto la TV durante 1 hora y ha jugado con un videojuego durante 45 minutos. El tiempo total es 1 hora + 45 minutos = 1 hora y 45 minutos.*

	Nada o menos de 10 minutos	Entre 11 y 30 minutos	Entre 31 y 59 minutos	Entre 1 hora y 1 hora y 29 minutos	Entre 1 hora y media y 2 horas	Más de 2 horas (escribir cuánto)
Lunes						...... horas y ...... minutos
Martes						...... horas y ...... minutos
Miércoles						...... horas y ...... minutos
Jueves						...... horas y ...... minutos
Viernes						...... horas y ...... minutos
Sábado						...... horas y ...... minutos
Domingo						...... horas y ...... minutos

PREGUNTAS DEL BLOQUE 2

Recuerde que para responder a estas preguntas debe preguntarle a su hijo/a. Si no está seguro de las respuestas, debe consultar la información con los profesores de la escuela.

Actividad física en la escuela

7. ¿Cuántas horas de Educación Física o actividades similares (por ejemplo, psicomotricidad, yoga, natación...) hacen a la semana en el curso de su hijo/a?

   En el curso de mi hijo/a hacen ……….. horas y ……..…. minutos de Educación Física a la semana.

8. ¿Su hijo/a realiza habitualmente las clases de Educación Física en la escuela?

   ☐ Mi hijo/a no hace Educación Física
   ☐ Mi hijo/a solo hace Educación Física cuando hay que hacer poco esfuerzo físico*
   ☐ Mi hijo/a siempre hace Educación Física

   * *Esfuerzo físico incluye todas las actividades que le hacen sudar y sentirse cansado o que hacen que se acelere su respiración como correr, saltar, hacer lanzamientos, practicar algún deporte….*

9. ¿Qué hace habitualmente su hijo/a durante el recreo en la escuela?

   ☐ Estar sentado (hablar, leer, jugar a juegos sin moverse, deberes…)
   ☐ Correr y jugar a juegos que impliquen poco esfuerzo físico*
   ☐ Correr y jugar intensamente todo el tiempo

Pausa a mediodía

10. ¿Qué hace habitualmente su hijo/a durante la pausa a mediodía (además de comer)? Responda a esta pregunta tanto si el niño se queda a comer en el comedor escolar como si va a comer a casa.

   ☐ Estar sentado (hablar, leer, jugar a juegos sin moverse, ver la TV, hacer deberes…)
   ☐ Correr y jugar a juegos que impliquen poco esfuerzo físico*
   ☐ Correr y jugar intensamente todo el tiempo

¿Estuvo enfermo su hijo/a la última semana o algo impidió que hiciera las actividades habituales?

☐ Sí
☐ No
